# Early Enteral Nutrition Tolerance in Patients With Cardiogenic Shock Requiring Mechanical Circulatory Support

**DOI:** 10.3389/fmed.2021.765424

**Published:** 2021-12-06

**Authors:** Wen-jun Liu, Jun Zhong, Jing-chao Luo, Ji-li Zheng, Jie-fei Ma, Min-jie Ju, Ying Su, Kai Liu, Guo-wei Tu, Zhe Luo

**Affiliations:** ^1^Department of Critical Care Medicine, Zhongshan Hospital, Fudan University, Shanghai, China; ^2^Department of Nursing, Zhongshan Hospital, Fudan University, Shanghai, China; ^3^Department of Critical Care Medicine, Xiamen Branch, Zhongshan Hospital, Fudan University, Xiamen, China; ^4^Shanghai Key Lab of Pulmonary Inflammation and Injury, Fudan University, Shanghai, China

**Keywords:** enteral nutrition, cardiogenic shock, mechanical circulatory support, vasoactive drugs, tolerance

## Abstract

**Background:** Enteral nutrition (EN) is recommended within the first 24–48 h for patients with hemodynamic stability, following admission to an intensive care unit (ICU). However, for patients with approximate stable hemodynamics requiring mechanical circulatory support and vasoactive drugs, the application of early EN remains controversial. We sought to evaluate the tolerance of early EN in patients with cardiogenic shock who required vasoactive drugs and mechanical circulatory support after cardiac surgery.

**Methods:** This single-center, prospective observational study included patients with cardiogenic shock, requiring vasoactive drugs and mechanical circulatory support after cardiac surgery, undergoing EN. The primary endpoint was EN tolerance and secondary endpoints were mortality, length of mechanical ventilation, and length of ICU stay.

**Results:** From February 2019 to December 2020, 59 patients were enrolled, of which 25 (42.37%) developed intolerance within 3 days of starting EN. Patients in the EN intolerant group had a longer median length of mechanical ventilation (380 vs. 128 h, *p* = 0.006), a longer median ICU stay (20 vs. 11.5 days, *p* = 0.03), and a higher proportion of bloodstream infections (44 vs. 14.71%, *p* = 0.018). The median EN calorie levels for all patients in the first 3 days of EN were 4.00, 4.13, and 4.28 kcal/kg/day, respectively. Median protein intake levels of EN in the first 3 days were 0.18, 0.17, and 0.17 g/kg/day, respectively. No significant difference was observed in the median dose of vasoactive drugs between the groups (0.035 vs. 0.05 μg/kg/min, *p* = 0.306).

**Conclusions:** Patients with cardiogenic shock after cardiac surgery had a high proportion of early EN intolerance, and patients with EN intolerance had a worse prognosis, but no significant correlation was identified between EN tolerance and the dose of vasoactive drugs.

## Introduction

According to the guidelines from the Society of Critical Care Medicine (SCCM) and the American Society for Parenteral and Enteral Nutrition (ASPEN), enteral nutrition (EN) is recommended for patients admitted to an intensive care unit (ICU) once hemodynamics are stable (1). Similarly, the European Society for Parenteral and Enteral Nutrition (ESPEN) guidelines also recommend that if oral intake is not possible, early EN (within 48 h) in critically ill adult patients should be performed/initiated rather than delaying it (2). Early EN nourishes the intestinal mucosa, maintains intestinal integrity, maintains intestinal microbial diversity, and improves immunity and metabolic function (3). Therefore, when compared with delayed EN, early EN reduces infectious complications (4–20).

Although early EN is recommended for most critically ill patients, the European Society of Intensive Care Medicine (ESICM) guidelines advocate seven scenarios that require delayed EN, including uncontrolled shock and failure to achieve hemodynamic and tissue perfusion targets (14). Some studies have demonstrated that vasoactive drugs aggravate visceral vasoconstriction and intestinal metabolic disorders caused by shock, and this may lead to intestinal ischemia (21). Similarly, early EN was speculated to cause abdominal distension, diarrhea, vomiting, aspiration, and possibly death (22–25). However, a recent study reported that intestinal ischemia is rare in patients receiving vasoactive drugs during EN; the incidence is 0.3–3.8% (21). However, while previous studies have focused on septic shock patients, data on EN safety in patients with cardiogenic shock are limited, and critically, conclusions are inconsistent (26–31). Furthermore, a consensus has not been reached on safe vasoactive drug doses during early EN.

Patients with cardiac surgery frequently present with circulatory failure for various reasons (32–35). Vasoactive drugs and mechanical circulatory support are thus required to achieve hemodynamic targets. In this study, we investigated the tolerance of early EN in patients taking vasoactive drugs and undergoing mechanical circulatory support after cardiac surgery to determine the effects of different vasoactive drug doses on the safety of early EN administration.

## Materials and Methods

### Study Design

This was a single-center prospective observational study. From February 2019 to December 2020, patients were continuously enrolled from a cardiac surgery ICU of a tertiary hospital. The ICU has 40 beds and accommodates various cardiac surgery perioperative patients. The study was approved by the ethics committee of Zhongshan Hospital, Fudan University (Approval No. B2019-075R), and patients or family members provided informed consent prior to study commencement.

### Participant Selection

Inclusion criteria were as follows: (1) age ≥ 18 years, (2) patients with cardiogenic shock receiving vasoactive drugs and mechanical circulatory support, including extracorporeal membrane oxygenation (ECMO) or intraaortic balloon pump (IABP), (3) mean arterial pressure ≥ 65 mmHg, (4) starting EN within 48 h after hemodynamic stability, and estimated EN duration ≥ 72 h.

Exclusion criteria included were as follows: (1) discontinued vasoactive drugs and mechanical circulatory support within 1 h after EN commencement, (2) situations where ESICM guidelines recommended EN should be delayed, i.e., uncontrolled hypoxemia and acidosis, uncontrolled gastric intestinal bleeding, overt bowel ischemia, bowel obstruction, abdominal compartment syndrome, and gastric aspirate volume > 500 ml/6 h. Cardiogenic shock was defined as a state of critical end-organ hypoperfusion due to reduced cardiac output (36).

### Data Collection and Outcome Definitions

According to the EN tolerance of patients, they were divided into EN tolerant and EN intolerant groups. EN intolerance was defined as gastric residual volume (GRV) > 250 ml on any day or any kind of EN complication (vomiting, abdominal distension, diarrhea, intestinal ischemia, and aspiration) within 3 days of EN (10, 12). Aspiration was defined as digestive fluid or EN solution in the respiratory tract by bronchoscope. Continuous gastrointestinal decompression was performed 1 h after the end of EN, and the amount of gastrointestinal decompression was defined as the GRV.

Patient baseline data were collected within 24 h after ICU admission, including patient characteristics, such as age, gender, height, weight, body mass index (BMI), types of mechanical circulatory support, comorbidity, previous cardiac surgery, left ventricular ejection fraction (LVEF) before surgery, acute physiology and chronic health evaluation (APACHE) II scores, surgery time, cardiopulmonary bypass time, and laboratory data such as liver function, renal function, cardiac biomarker, and serum lactate indices. Nutrition-related data were collected for 3 consecutive days after the start of EN and included daily GRV, EN volume, protein levels, calories provided by EN, calories provided by parenteral nutrition (PN), and calories provided by propofol. These data were derived from the in-house electronic medical record system and nurse-record sheets.

Information on vasoactive drug types and doses in the first 3 days of EN were collected. The following formula was used to calculate the equivalent dose of norepinephrine, where equivalent dose of norepinephrine = norepinephrine (μg/min) + dopamine (μg/min) ÷ 2 + epinephrine (μg/min) + phenylephrine (μg/min) ÷ 10 + vasopressin (U/h) × 8.33 (26). Based on previous practices, according to vasoactive drug doses, patients were divided into low- and high-dose groups: the average equivalent dose of norepinephrine <0.1 μg/kg/min during EN was defined as the low-dose group, whereas ≥0.1 μg/kg/min was defined as the high-dose group (27).

The primary study endpoint was patient EN intolerance. Secondary endpoints were the length of mechanical ventilation, length of ICU stay, length of stay in the hospital, in-hospital mortality, the incidence of infection, and the site of infection (e.g., pulmonary, wound, urinary system, and bloodstream infection). Intolerance signs were also recorded, including GRV > 250 ml and EN-related adverse events (e.g., vomiting, abdominal distension, diarrhea, intestinal ischemia, and aspiration).

### Statistical Analysis

All statistical analyses were performed using IBM SPSS Statistics version 20. After using the KS test to evaluate data normality, normally distributed data were represented by the mean (±SD), and non-normally distributed data were represented by the median [interquartile range (IQR)]. The χ^2^ -test was used for categorical variables and the *t*-test or Mann–Whitney *U*-test for continuous variables. A *p* < 0.05 value was considered statistically significant.

## Results

### Baseline Patient Characteristics

**Figure 1:**
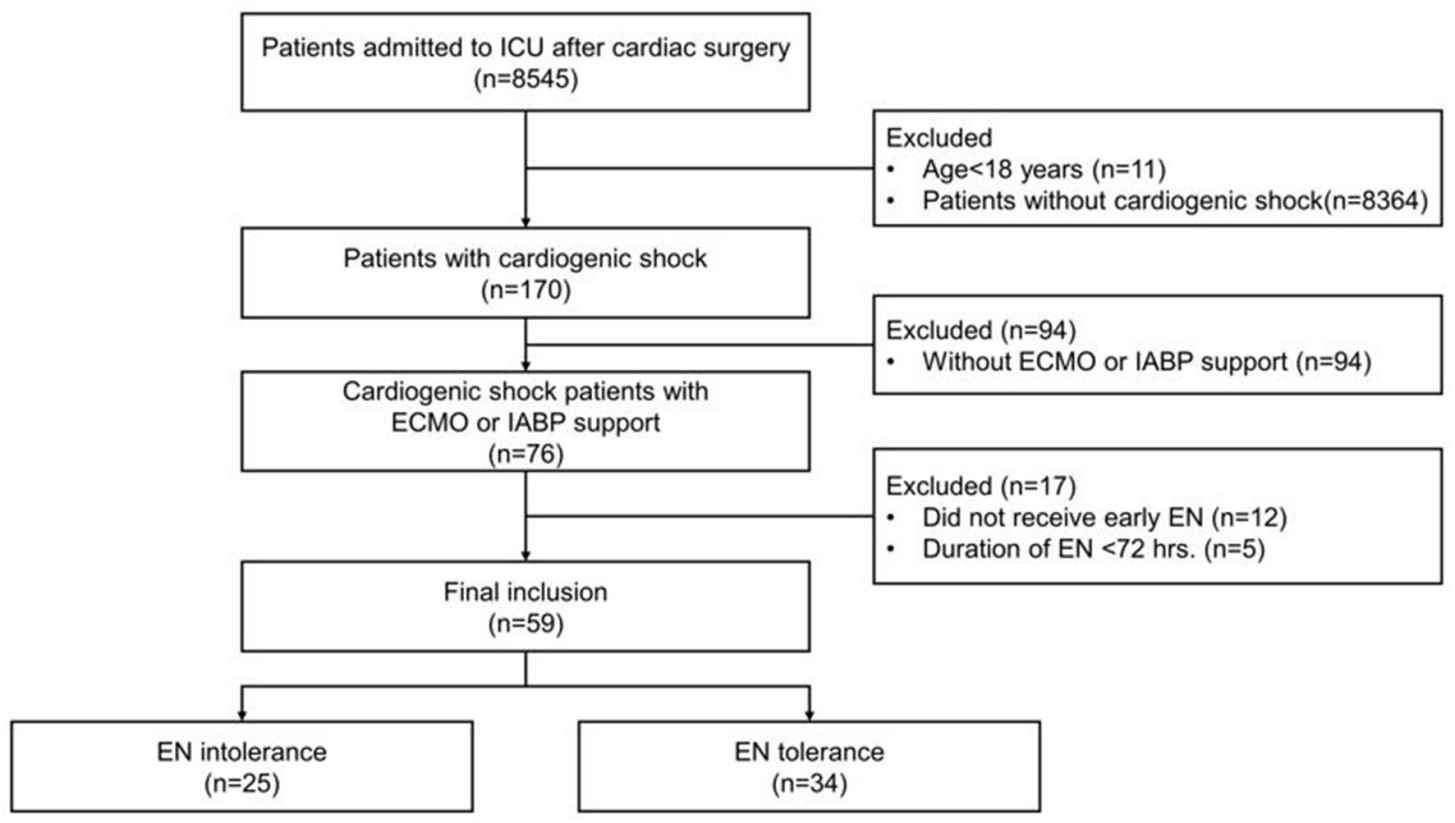
Flowchart of patient selection. ICU, intensive care unit; EN, enteral nutrition.

From February 2019 to December 2020, 8,545 patients, after cardiac surgery, were admitted to the cardiac surgery ICU. In total, 170 adult patients were diagnosed with cardiogenic shock with an ICU stay >72 h. Of these, 94 patients were excluded because they did not receive mechanical circulatory support, 12 were excluded because they did not receive early EN, and five were excluded because the EN duration was <72 h. Therefore, 59 patients were finally enrolled and divided into two groups; 25 in the EN intolerant group and 34 in EN tolerant group (Figure 1).

Patient baseline data are shown in Table 1. The median age was 63 years old, the majority were male (77.97%), and the average BMI was 23.5 kg/cm^2^. Sixteen patients received ECMO, 46 received IABP, and 10 had received cardiac surgery prior to this admission. The median APACHE II score was 10, the average LVEF before surgery was 49%, the median surgery time was 293 min, the median cardiopulmonary bypass time was 137 min, the median cardiac troponin T (cTnT) level was 1 ng/mL, the median N-terminal probrain natriuretic peptide (NT-proBNP) level was 2,994 pg/mL, and the median lactate level was 4.6 mmol/L. The proportion of men in the EN intolerant group was significantly higher (92 vs. 68%, *p* = 0.03). In the EN intolerant group, the proportion of patients receiving ECMO was higher (48 vs. 11.76%, *p* = 0.003). We observed no statistical differences between groups in terms of age, BMI, comorbidity, pre-operative cardiac functions, APACHE II scores, surgery time, cardiopulmonary bypass time, and indices for liver function, renal function, cardiac biomarkers, and serum lactate levels.

**Table 1:** Patient baseline characteristics grouped by EN intolerance or tolerance.

**Characteristics**	**Total (*n* = 59)**	**Intolerance (*n* = 25)**	**Tolerance (*n* = 34)**	***P*** **-value**
Age (y)	63 (55–67)	62 (52–66)	63 (55–68)	0.628
Gender (male, %)	46 (77.97)	23 (92.00)	23 (67.65)	0.030
Weight (kg)	65 (60–75)	65 (62–73)	64 (60–79)	0.718
Height (cm)	168.93 ± 8.70	172.04 ± 7.74	166.65 ± 8.77	0.020
BMI (kg/cm^2^)	23.54 ± 4.41	22.67 ± 4.04	24.17 ± 4.62	0.425
ECMO (*n*, %)	16 (27.12)	12 (48.00)	4 (11.76)	0.003
IABP (*n*, %)	46 (78.00)	14 (56.00)	32 (94.10)	0.001
LVEF before surgery (%)	49.05 ± 13.88	51.36 ± 12.35	47.35 ± 14.85	0.326
APACHE II	10 (7–18)	13 (7–19)	9.5 (7–13)	0.177
Surgery time (min)	293 (219–380)	277 (178–395)	305 (240.5–365)	0.179
Cardiopulmonary bypass time (min)	137 (0–191)	130 (0–207)	146 (48–187)	0.895
TBil (μmol/L)	13.89 ± 7.60	15.49 ± 8.04	12.72 ± 7.14	0.187
Total protein (g/L)	65.42 ± 6.28	63.80 ± 7.35	66.62 ± 5.15	0.112
Albumin (g/L)	39.37 ± 4.49	38.24 ± 5.20	40.21 ± 3.76	0.113
AST (U/L)	23 (14–36)	23 (14–35)	24 (14–38)	0.794
ALT (U/L)	23 (17–36)	24 (18–36)	22 (17–36)	0.908
cTnT (ng/ml)	1 (0.50–2.73)	0.86 (0.52–2.72)	1.61 (0.47–3.55)	0.634
NT-proBNP (pg/ml)	2,994 (1,410–8,199)	2,914 (1,322–6,293)	3,239.5 (1,606–9,757)	0.586
Lactate (mmol/L)	4.6 (1.5–8.3)	4.6 (1.4–9.0)	4.55 (1.6–7.7)	0.914
eGFR (ml/min/1.73 m^2^)	68 (62–89)	65 (61–81)	71 (63–90)	0.205
Hypertension (*n*, %)	29 (49.15)	13 (52.00)	16 (47.06)	0.795
Diabetes mellitus (*n*, %)	14 (23.73)	6 (24.00)	8 (23.53)	1.000
CKD (*n*, %)	7 (11.86)	5 (20.00)	2 (5.88)	0.122
Cerebral infarction (*n*, %)	6 (10.17)	3 (12.00)	3 (8.82)	0.691
Myocardial infarction (*n*, %)	2 (3.39)	1 (4.00)	1 (2.94)	1.000
Cardiac surgery (*n*, %)	10 (16.95)	4 (16.00)	6 (17.65)	1.000

*EN, enteral nutrition; BMI, body mass index; CKD, chronic kidney disease; LVEF, left ventricular ejection fraction; APACHE II, Acute Physiology and Chronic Health Evaluation II; ICU, intensive care unit; TBil, total bilirubin; ALT, alanine transaminase; AST, aspartate transaminase; cTnT, cardiac troponin T; NT-proBNP, N-terminal pro brain natriuretic peptide; eGFR, estimated glomerular filtration rate.*

*Categorical variables are expressed as n (%). Continuous variables are expressed as mean ± SD or median (Q1–Q3)*.

### Primary and Secondary Outcomes

As indicated, 25 (42.37%) patients were EN intolerant. As shown in Table 2, the median dose of norepinephrine equivalent in EN tolerant and intolerant groups was not statistically different (0.035 vs. 0.05 μg/kg/min, *p* = 0.306). The median length of mechanical ventilation in the EN intolerant group was significantly longer (380 vs. 128 h, *p* = 0.006). In addition, the median length of ICU stay for the EN intolerant group was longer than the EN tolerant group (20 vs. 11.5 days, *p* = 0.03), but no significant difference in the length of hospital stay was observed between the groups (31 vs. 25.5 days, *p* = 0.519). Although the mortality rate between groups was not statistically different, the mortality rate of the EN intolerant group was almost twice that of the EN tolerant group (40 vs. 20.59%, *p* = 0.147). Moreover, the proportion of patients receiving CRRT was slightly higher in the EN intolerant group (36 vs. 17.65%, *p* = 0.137).

**Table 2:** The primary and secondary outcomes, grouped by EN intolerance or tolerance.

**Characteristics**	**Total**	**Intolerance**	**Tolerance**	***P-*** **value**
	**(*n* = 59)**	**(*n* = 25)**	**(*n* = 34)**	
Norepinephrine equivalents (μg/min)	3.17 (1.33–7.14)	3.83 (2.42–6.57)	2.25 (1.13–10.96)	0.330
Norepinephrine equivalents (μg/kg/min)	0.04 (0.02–0.10)	0.05 (0.04–0.09)	0.03 (0.01–0.135)	0.306
Initial dose of EN (mL)	200 (200–450)	250 (200–500)	200 (200–300)	0.234
Average dose for the first 3 days of EN (mL)	317 (233–450)	256.67 (233.33–458.34)	316.67 (224.17–412.50)	0.829
CRRT (*n*, %)	15 (25.4)	9 (36.0)	6 (17.6)	0.137
Length of mechanical ventilation (h)	216 (77–408)	380 (102–576)	128 (58.5–300)	0.006
Length of ICU stay (day)	16 (7–25)	20 (11–31)	11.5 (7–18)	0.030
Length of hospital stay (day)	28 (20–43)	31 (19–46)	25.5 (20–37)	0.519
Mortality (*n*, %)	17 (28.81)	10 (40.00)	7 (20.59)	0.147
Infection rate (*n*, %)	40 (67.80)	20 (80.00)	20 (58.82)	0.100
Pulmonary infection (*n*, %)	39 (66.10)	20 (80.00)	19 (55.88)	0.094
Wound infection (*n*, %)	2 (3.39)	1 (4.00)	1 (2.94)	1.000
Urinary tract infection (*n*, %)	2 (3.39)	0	2 (5.89)	0.503
Bloodstream infection (*n*, %)	16 (27.12)	11 (44.00)	5 (14.71)	0.018

*EN, enteral nutrition; ECMO, extracorporeal membrane oxygenation; IABP, intra-aortic balloon pump; CRRT, continuous renal replacement therapy; ICU, intensive care unit.*

*Categorical variables are expressed as n (%). Continuous variables are expressed as mean ± SD or median (Q1–Q3)*.

We observed no statistical difference in the dose of enteral feeding on the first 3 days between the groups. In terms of post-operative infections, the bloodstream infection rate in the EN intolerant group was higher than the EN tolerant group (44 vs. 14.71%, *p* = 0.018). Pulmonary infection was more common in the EN intolerant group, although no statistical differences were identified between the groups (80 vs. 55.88%, *p* = 0.094).

Among EN intolerant patients, nine (36%) had GRV > 250 mL, seven (28%) experienced aspiration, six (24%) had diarrhea, three (12%) experienced vomiting, and five (20%) had abdominal distension. No bowel ischemia occurred in this study (Table 3).

**Table 3:** Signs for intolerance (*N* = 25).

**Signs for intolerance**	***N*** **(%)**
GRV > 250 mL	9 (36)
Aspiration	7 (28)
Diarrhea	6 (24)
Vomiting	3 (12)
Abdominal distention	5 (20)
Intestinal ischemia	0 (0)

*GRV, gastric residual volume.*

*Variables are expressed as n (%)*.

The calorie and protein intake of all patients in the first 3 days are shown (Table 4). The median calorie levels of EN in the first 3 days were 4.00, 4.13, and 4.28 kcal/kg/day, respectively, with an upward trend each day. The median calorie levels of PN in the first 3 days were 7.28, 6.55, and 6.10 kcal/kg/day, respectively, indicating a daily downward trend. The median total calories in the first 3 days were 10.87, 10.84, and 10.28 kcal/kg/day, respectively, which were basically the same. The median EN volume for patients in the first 3 days was 200, 280, and 400 mL, respectively, indicating an increasing trend. The median protein intake of EN in the first 3 days was 0.18, 0.17, and 0.17, respectively, whereas the median total protein intake was 0.61, 0.73, and 0.57 g/kg/day, respectively (Table 4). However, no significant differences were observed in either calorie or protein intake between the groups.

**Table 4:** Patients' energy and protein intake in the first 3 days.

**Characteristics**	**Day 1**				**Day 2**				**Day 3**			
	**Total**	**EN intolerance**	**EN tolerance**	***P-*** **value**	**Total**	**EN intolerance**	**EN tolerance**	***P-*** **value**	**Total**	**EN intolerance**	**EN tolerance**	***P-*** **value**
Calorie received from EN (kcal/kg/day)	4.00 (2.92–5.00)	4 (0.85–6.93)	4 (2.97–4.35)	0.618	4.13 (1.56–7.52)	3.47 (0.77–7.51)	4.21 (2.09–7.81)	0.228	4.28 (2.53–7.00)	3.88 (2.14–7.69)	4.52 (2.51–6.80)	0.976
Calorie received from propofol (kcal/kg/day)	0 (0–2.00)	0 (0–1.67)	0.48 (0–2.15)	0.226	0 (0–0.59)	0 (0–0)	0 (0–1.42)	0.099	0 (0–0.35)	0 (0–0.65)	0 (0–0.45)	0.916
Calorie received from PN (kcal/kg/day)	7.28 (5.88–8.85)	6.52 (6.01–9.05)	7.50 (5.67–8.78)	0.866	6.55 (5.31–8.35)	6.74 (5.66–8.19)	6.38 (4.79–8.43)	0.434	6.10 (4.40–7.52)	6.55 (4.78–7.55)	6.09 (3.75–7.51)	0.576
Total calorie received (kcal/kg/day)	10.87 (9.47–13.27)	11.87 (8.74–16.30)	12.18 (10.01–14.88)	0.963	10.84 (8.10–14.91)	10.16 (8.08–14.87)	12.14 (8.59–15.70)	0.510	10.28 (7.2–13.67)	12.35 (7.01–15.12)	10.24 (7.05–15.02)	0.794
Dose of EN (mL)	200 (200–450)	250 (200–500)	200 (200–300)	0.234	280 (200–500)	250 (200–500)	350 (237.5–500)	0.241	400 (200–500)	450 (200–500)	400 (200–500)	0.691
Protein received from EN (g/kg/day)	0.18 (0.13–0.21)	0.18 (0–0.27)	0.18 (0.13–0.2)	0.536	0.17 (0–0.29)	0.16 (0–0.29)	0.19 (0–0.32)	0.476	0.17 (0–0.28)	0.17 (0–0.30)	0.16 (0–0.28)	0.771
Total protein received (g/kg/day)	0.61 (0.48–0.80)	0.65 (0.53–0.88)	0.59 (0.39–0.76)	0.160	0.73 (0.49–0.92)	0.74 (0.56–0.92)	0.67 (0.38–0.95)	0.386	0.57 (0.33–0.85)	0.58 (0.57–0.99)	0.56 (0.32–0.85)	0.490

*EN, enteral nutrition; PN, parenteral nutrition.*

*Continuous variables are expressed as median (Q1–Q3)*.

According to vasoactive drug doses, the average equivalent dose of norepinephrine <0.1 μg/kg/min was defined as the low-dose group, whereas norepinephrine ≥0.1 μg/kg/min was defined as the high-dose group. Surgery time (278.5 vs. 443.0 min, *p* = 0.01) and cardiopulmonary bypass time (115.5 vs. 174.0 min, *p* = 0.04) were significantly shorter in the low-dose group. Levels of cTnT after surgery in the low-dose group were lower (0.84 vs. 2.16 ng/mL, *p* = 0.005), and NT-proBNP levels in the low-dose group exhibited a lower trend (2,889.5 vs. 4,714 pg/mL, *p* = 0.247). Serum lactate levels after surgery in both the groups were not significantly different (3.55 vs. 5.30 mmol/L, *p* = 0.37).

**Figure 2:**
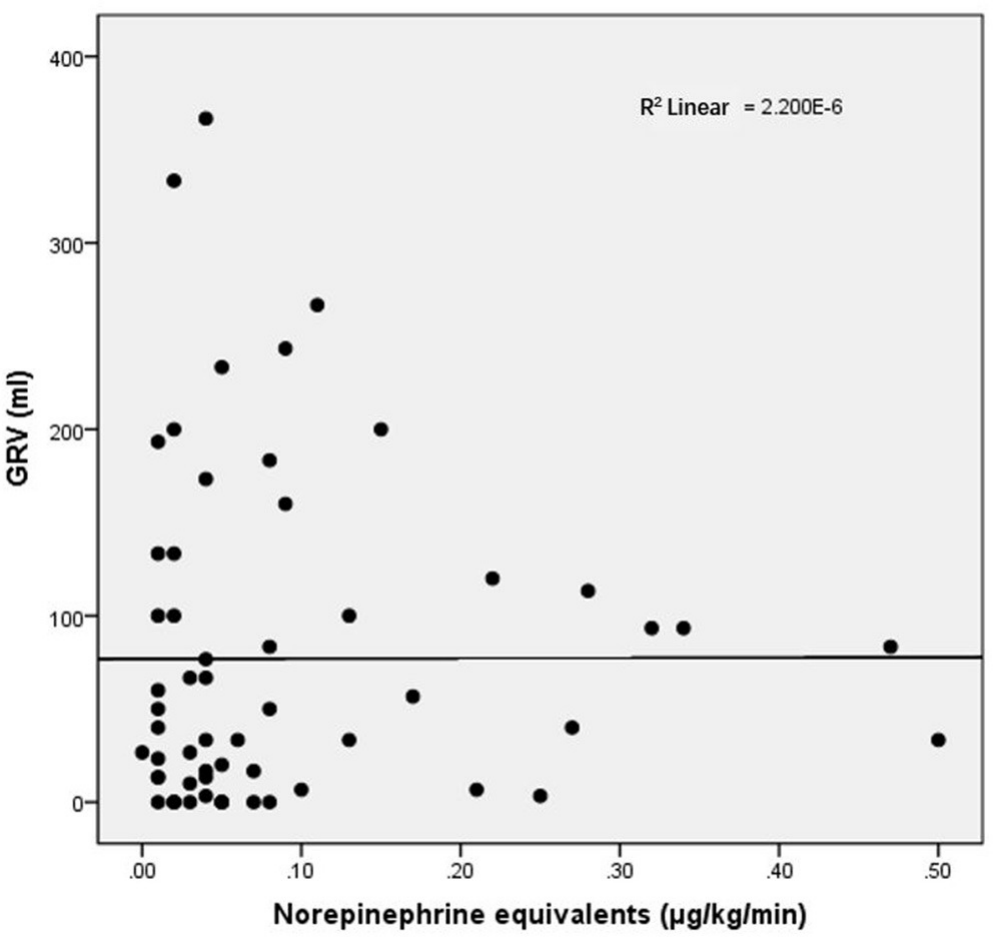
Relationship between vasoactive drug dosage and GRV. GRV, gastric residual volume.

For other baseline data, no significant differences were recorded between the groups (Table 5). Clinical outcomes are shown in Table 6. The CRRT rate in the high-dose group was higher (60 vs. 13.6%, *p* = 0.001). The length of mechanical ventilation tended to be prolonged in the high-dose group (348 vs. 150 h, *p* = 0.095), and the mortality rate also tended to increase in the high-dose group (46.7 vs. 22.7%, *p* = 0.078). No significant difference in GRV was observed between high- and low-dose groups (83.33 vs. 33.33 mL, *p* = 0.235). As shown in Figure 2, the equivalent dose of norepinephrine and GRV was discretely distributed in the scatter plot, and *R*^2^ was 2.200 × 10^−6^, which indicated no correlation between the equivalent dose of norepinephrine and GRV. No significant difference was observed in the EN intolerance rates between high- and low-dose groups (33.3 vs. 45.5%, *p* = 0.305).

**Table 5:** Patient baseline characteristics, grouped by the dose of norepinephrine equivalents.

**Characteristics**	**Low dose** **(<0.1 μg/kg/min)** ***n*** **= 44**	**High dose** **(≥0.1 μg/kg/min)** ***n*** **= 15**	***P-*** **value**
Age (y)	62.50 (55.25–67.75)	64.00 (48.00–65.00)	0.507
Gender (male, %)	36 (81.8)	10 (66.7)	0.192
Weight (kg)	65.00 (60.00–75.00)	65.00 (55.00–74.00)	0.656
Height (cm)	168.95 ± 8.03	168.87 ± 10.77	0.818
BMI (kg/cm^2^)	23.57 ± 4.11	23.42 ± 5.35	0.108
LVEF before surgery (%)	47.36 ± 13.80	54.00 ± 13.33	0.848
ECMO (*n*, %)	14 (31.8)	2 (13.3)	0.145
IABP (*n*, %)	33 (75)	13 (86.7)	0.290
APACHE II	10.00 (7.00–14.75)	13.00 (9.00–22.00)	0.074
Surgery time (min)	278.50 (219.00–327.50)	443.00 (293.99–600.00)	0.010
Cardiopulmonary bypass time (min)	115.50 (0–188.00)	174.00 (132.00–367.00)	0.040
TBil (μmol/L)	13.86 ± 7.58	13.99 ± 7.91	0.652
Total protein (g/L)	65.36 ± 5.98	65.60 ± 7.32	0.752
Albumin (g/L)	39.48 ± 4.24	39.48 ± 4.24	0.595
AST (U/L)	25.00 (14.50–36.00)	21.00 (14.00–45.00)	0.571
ALT (U/L)	23.50 (17.00–39.75)	22.00 (19.00–26.00)	0.423
cTnT (ng/ml)	0.84 (0.35–2.44)	2.16 (1.00–6.00)	0.005
NT-proBNP (pg/ml)	2,889.50 (1,338.00–6,776.50)	4,714.00 (1,654.00–18,221.00)	0.247
Lactate (mmol/L)	3.55 (1.53–7.48)	5.30 (1.50–10.70)	0.370
eGFR (ml/min/1.73 m^2^)	68 (62–89)	66 (60–78)	0.338
Hypertension (*n*, %)	19 (43.2)	10 (66.7)	0.101
Diabetes mellitus (*n*, %)	13 (29.5)	1 (6.7)	0.067
CKD (*n*, %)	4 (9.1)	3 (20.0)	0.243
Cerebral infarction (*n*, %)	3 (6.8)	3 (20.0)	0.165
Myocardial infarction (*n*, %)	0	2 (13.3)	0.061
Cardiac surgery (*n*, %)	6 (13.6)	4 (26.7)	0.218

*EN, enteral nutrition; BMI, body mass index; CKD, chronic kidney disease; LVEF, left ventricular ejection fraction; APACHE II, Acute Physiology and Chronic Health Evaluation II; ICU, intensive care unit; TBil, total bilirubin; ALT, alanine transaminase; AST, aspartate transaminase; cTnT, cardiac troponin T, NT-proBNP, N-terminal pro brain natriuretic peptide; eGFR, estimated glomerular filtration rate.*

*Categorical variables are expressed as n (%). Continuous variables are expressed as mean ± SD or median (Q1–Q3)*.

**Table 6:** The primary and secondary outcome, grouped by the dose of norepinephrine equivalents.

**Characteristics**	**Low dose**	**High dose**	***P-*** **value**
	**(<0.1 μg/kg/min)**	**(≥0.1 μg/kg/min)**	
	***n*** **= 44**	***n*** **= 15**	
Norepinephrine equivalents (μg/min)	2.25 (1.05–3.79)	14.00 (10.39–20.49)	<0.001
Norepinephrine equivalents (μg/kg/min)	0.04 (0.01–0.05)	0.22 (0.13–0.32)	<0.001
Enteral nutritional intolerance (*n*, %)	20 (45.5)	5 (33.3)	0.305
GRV (mL)	33.33 (5.00–125.00)	83.33 (33.33–113.33)	0.235
Initial dose of EN (mL)	200.00 (200.00–487.50)	200.00 (200.00–250.00)	0.783
Average dose for the first 3 days of EN (mL)	320.00 (228.34–487.50)	316.67 (233.33–350.00)	0.643
CRRT (*n*, %)	6 (13.6)	9 (60)	0.001
Length of mechanical ventilation (h)	150 (73.25–393.00)	348.00 (132.00–456.00)	0.095
Length of ICU stay (day)	15.00 (7.00–23.75)	17.00 (10.00–26.00)	0.276
Length of hospital stay (day)	27.00 (20.00–41.00)	30.00 (20.00–47.00)	0.464
Mortality (*n*, %)	10 (22.7)	7 (46.7)	0.078
Infection rate (*n*, %)	30 (68.2)	10 (66.7)	0.576
Pulmonary infection (*n*, %)	29 (65.9)	10 (66.7)	0.609
Wound infection (*n*, %)	1 (2.3)	1 (6.7)	0.447
Urinary tract infection (*n*, %)	1 (2.3)	1 (6.7)	0.447
Bloodstream infection (*n*, %)	12 (27.3)	4 (26.7)	0.623

*EN, enteral nutrition; ECMO, extracorporeal membrane oxygenation; IABP, intra-aortic balloon pump; CRRT, continuous renal replacement therapy; ICU, intensive care unit.*

*Categorical variables are expressed as n (%). Continuous variables are expressed as median (Q1–Q3)*.

## Discussion

In our study, 25/59 patients (42.37%) with cardiogenic shock after cardiac surgery were intolerant to EN, similar to previous studies (26–28). Merchan et al. reported for patients with sepsis receiving vasoactive drugs that 62/120 were EN tolerant, indicating an intolerance rate of 48.33% (26). However, in another study, 259 patients with vasoactive drug support had an EN intolerance rate of 25.1% (27). For patients undergoing mechanical ventilation, a previous study with 1,888 ICU patients showed an EN intolerance rate of 30.5% (28). Thus, differences in EN intolerance rates between these studies may have arisen due to different patient populations and non-uniform definitions of EN intolerance. In patients who received mechanical ventilation >72 h after cardiovascular surgery, an EN intolerance rate of 43.68% was determined (28), similar to our data. This result suggested that patients with cardiogenic shock had a higher rate of EN intolerance during vasoactive drug and mechanical circulatory support. Therefore, caution should be exercised when these patients commence EN.

It was previously demonstrated that patients with EN intolerance had higher mortality, shorter length of mechanical ventilation free time, longer ICU stays, reduced calorie intake, and poorer outcomes (26, 28). Our study confirmed these findings. Patients with EN intolerance had significantly longer mechanical ventilation time, longer ICU stays, and a higher incidence of bloodstream infections. Although no statistical differences in mortality were observed between groups, mortality in the EN intolerant group was approximately twice that of the EN tolerant group. Our research demonstrated that 28% of EN intolerant patients experienced aspiration issues, which may be partially explained by the high incidence of pulmonary infections in these patients. Unfortunately, there was no statistical difference in mortality due to the small sample size.

In previous studies, the caloric compliance rate of EN in some patients with shock was between 40 and 89.8% (28, 29), among which EN intolerant patients received approximately 10.9–12 kcal/kg/d (26, 27). Our ICU had previously adopted a trophic nutrition strategy for patients with cardiogenic shock. Both calorie and protein intake levels were lower than previous studies. This may have been due to the high rate of EN intolerance in this study, and also the requirement for patients with cardiogenic shock to be fluid restricted. Hence, calorie and protein nutrition from EN and PN were both low. Furthermore, based on our previous findings, the implementation of a soybean-based intravenous fat emulsion restriction diet in cardiac surgical patients was associated with a reduced post-operative nosocomial infection rate (37). It also reduced the length of ICU/hospital stay, hospital costs, mechanical ventilation time, and a lower incidence of cholestasis. Therefore, our cardiovascular center implemented a soybean-based intravenous fat emulsion restriction diet for cardiac surgical patients. This factor was the cause of relatively low calorie and protein levels.

The relationship between vasoactive drug doses and tolerance and prognosis of early EN is inconsistent in the literature. Mancl et al. reported that in patients with septic shock, the incidence of EN intolerance was positively correlated with vasoactive drug doses (27). Therefore, many studies have sought to determine safe vasoactive drug doses when implementing early EN. Ohbe et al. compared differences in clinical outcomes for early (<48 h) or late (≥48 h) EN in shock patients on mechanical ventilation taking vasoactive drugs. Patients were divided into three groups based on the norepinephrine equivalent: low (<0.1 μg/kg/min), medium (0.1–0.3 μg/kg/min), and high (>0.3 μg/kg/min) doses. The 28-day mortality rate was significantly lower in the early EN than in the late EN groups in low- and medium-dose groups. In the high-dose group, the 28-day mortality rate did not differ significantly between the early EN and the late EN groups. Additionally, no significant difference was observed in the non-obstructive mesenteric ischemia rate (0.2 vs. 0.3%) between the early and the late EN groups (28). Thus, when supported by low and medium vasoactive drug doses, early EN was considered safe and it improved patient outcomes. Another study revealed it was safe to commence early EN in mechanically ventilated septic shock patients, with norepinephrine usage <0.14 μg/kg/min (26). However, early EN is not always safe for patients taking vasoactive drugs and those who are on mechanical ventilation support. Reignier et al., compared patient outcomes in those receiving EN and PN who were under mechanical ventilation and vasoactive drug support. Although no differences in mortality were determined, intestinal ischemia was significantly increased in patients on EN. It should be noted these patients received a very high vasopressor dose (average 0.53 μg/kg/min) (21). This observation suggested that when high-dose vasoactive drugs are used, EN should be administered with caution.

In our study, based on the vasoactive drug dose, the EN intolerance rate was 45.5% in the low-dose group and 33.3% in the high-dose group. Furthermore, no significant correlation between the average GRV and vasoactive drug dose was observed. This result was inconsistent with previous studies (28) and maybe related to our small sample size. Previous studies reported it was unsafe to commence EN when high-dose vasoactive drugs were used, and the rate of EN tolerance was negatively correlated with the dose of vasoactive drugs. The reported safe dosage is <0.14–0.32 μg/kg/min (26, 31). In our study, the overall vasoactive drug dose was relatively low, and most patients were within safe doses, as reported previously. This possibly explained the non-significant correlation between the average GRV and the vasoactive drug dose. In addition, serum lactate levels in low- and high-dose groups exhibited no statistical differences, suggesting that the timing of EN initiation cannot be based only on the vasoactive drug dose and serum lactate levels. Thus, the optimal timing for EN initiation requires further investigation.

Our study had some limitations. First, the sample size was small, with only 59 patients; thus, some bias may have been introduced. Second, the overall dose of vasoactive drugs was low, and most were within safe doses as indicated by previous studies; however, this factor may have restricted analyses of the relationships between vasoactive drug doses and the rate of EN intolerance. Third, patient calorie and protein intakes were low, which may have been related to fluid restriction and the high EN intolerant rate generated by cardiogenic shock.

## Conclusions

Patients with cardiogenic shock, taking vasoactive drugs, and undergoing mechanical circulatory support had a high proportion of early EN intolerance, which was associated with adverse prognoses. However, no significant correlations were identified between EN tolerance and vasoactive drug doses.

## Data Availability Statement

The original contributions presented in the study are included in the article/supplementary material, further inquiries can be directed to the corresponding authors.

## Ethics Statement

The studies involving human participants were reviewed and approved by Ethics Committee of Zhongshan Hospital, Fudan University. The patients/participants provided their written informed consent to participate in this study.

## Author Contributions

W-jL, JZ, G-wT, and ZL: conception and design. G-wT and ZL: administrative support. JZ, J-cL, and J-fM: collection and assembly of data. W-jL and JZ: data analysis and interpretation. All authors: manuscript writing and final approval of manuscript.

## Funding

This study was supported by the Construction Program of key but weak disciplines of Shanghai Health Commission (2019ZB0105), Natural Science Foundation of Shanghai (20ZR1411100 and 21ZR1412900), Science and Technology Commission of Shanghai Municipality (20DZ2261200), National Natural Science Foundation of China (82070085), Clinical Research Project of Zhongshan Hospital (2020ZSLC38 and 2020ZSLC27), Smart Medical Care of Zhongshan Hospital (2020ZHZS01), Project for the elite backbone of Zhongshan Hospital (2021ZSGG06).

## Conflict of Interest

The authors declare that the research was conducted in the absence of any commercial or financial relationships that could be construed as a potential conflict of interest.

## Publisher's Note

All claims expressed in this article are solely those of the authors and do not necessarily represent those of their affiliated organizations, or those of the publisher, the editors and the reviewers. Any product that may be evaluated in this article, or claim that may be made by its manufacturer, is not guaranteed or endorsed by the publisher.
